# Psychotropic Polypharmacy and QT Prolonging Medications in Hospitalized Patients

**DOI:** 10.1002/prp2.70107

**Published:** 2025-04-29

**Authors:** Joel Moore, Isabella Singh, Ruby Tszwai Au, Genevieve Gabb, Joanne Eng‐Frost, Elizabeth Hotham, Sepehr Shakib, Vijayaprakash Suppiah

**Affiliations:** ^1^ UniSA Clinical and Health Sciences University of South Australia Adelaide South Australia Australia; ^2^ Department of Cardiovascular Medicine Flinders Medical Centre Bedford Park South Australia Australia; ^3^ College of Medicine & Public Health, Flinders University of South Australia Bedford Park South Australia Australia; ^4^ Department of Clinical Pharmacology The Royal Adelaide Hospital Adelaide South Australia Australia; ^5^ Discipline of Pharmacology School of Medicine, University of Adelaide Adelaide South Australia Australia; ^6^ Australian Centre for Precision Health, University of South Australia Adelaide South Australia Australia

## Abstract

It is common for patients with mental illnesses to be prescribed multiple psychotropic medications to effectively manage their conditions. Psychotropic polypharmacy has been shown to potentiate and increase the risks of several adverse effects, including QT prolongation. This study aimed to investigate the prescribing trends of and differences in prescribing of QT‐prolonging medications (QTPMs) at admission and discharge in hospitalized patients. This retrospective observational study utilized inpatient data from three public hospitals between January and December 2019. QTPMs were classified according to the AZCERT classification. QTPMs doses were evaluated by calculating the ratio of prescribed daily dose (PDD) to the defined daily dose (DDD). Subgroup analyses showed significant differences between patient groups on admission and discharge (all *p* < 0.001). Mean QTPMs decreased significantly between the two time points only in patients admitted to acute medical and geriatric units (*p* < 0.001). PDD/DDD ratio for conditional risk QTPMs in acute mental health unit (AMHU) patients was increased at discharge (*p* = 0.038). Patients admitted to acute medical and geriatric units were four and eight times more likely to be discharged with one QTPM with known risk in combination with more QTPMs with conditional risk. Logistic regression showed significant relationships with age and total number of regular medicines at admission for those prescribed high‐dose QTPMs at discharge. The findings underscore the necessity for enhanced monitoring of QTPMs in hospitalized patients, particularly for those at higher risk.

## 1 Introduction

Psychotropic polypharmacy is commonly experienced by patients with mental illnesses, increasing their risk of adverse effects when compared with the general population [1, 2]. Adverse effects of psychotropic medications are wide‐ranging, including increased sedation, salivary hypersecretion, and akathisia [3]. Psychotropic medications can also induce serious cardiac complications, including QT interval prolongation [4]. The QT interval represents the period between ventricular depolarization and repolarization. Corrected QT interval (QTc) values account for the variation of QT interval with heart rate. QTc is prolonged if it is greater than 440 ms in men or greater than 460 ms in women [5]. QTc prolongation increases the risk of Torsades de Pointes (TdP), ventricular tachyarrhythmic syncope, and sudden cardiac death, with QT intervals > 500 ms positively correlated with a higher risk of cardiac arrhythmias [2, 6]. Non‐modifiable risk factors for QT prolongation include advanced age, female sex, genetic predisposition for long QT syndrome, and structural heart disease (e.g., bileaflet mitral valve prolapse and infiltrative cardiomyopathies) [4].

The Arizona Center for Education and Research on Therapeutics (AZCERT) classification has classified QT prolonging medications (QTPMs) into three categories [7]. Medications in this classification have ‘known’, ‘possible’ or ‘conditional’ risks. In the first category, medications frequently cause QTc prolongation and in a dose‐dependent manner cause TdP. Medications of the second class frequently prolong QTc but rarely cause TdP and the last category consists of medications whose risk of causing QTc prolongation and TdP depends on the presence of essential cofactors such as hypokalemia, promoting the adverse reaction. Previous study had shown that combinations of medications with ‘possible’ risk had no additional risk of QTc prolongation. However, the risk of QTc prolongation appeared additive with increasing the number of medications of the ‘conditional’ and ‘known’ risk classes, with the magnitude being dependent on the presence of further risk factors [8].

In‐hospital prescribing of medications with the additive risk of QT prolongation remains a significant and modifiable risk factor, especially in vulnerable populations including elderly patients and patients with mental health illnesses. Given the paucity of literature surrounding the in‐hospital prescribing trends of QTPMs, this study aimed to explore the prescribing trends of and differences in QTPMs prescribing between admission and discharge in subgroups of patients across South Australian public hospitals.

## 2 Method

Data of patients taking at least one antipsychotic medication prior to admission from three South Australian tertiary public hospitals between 1 January and 31 December 2019 were obtained from electronic medical records as described elsewhere [9]. All medications were categorized as having known, possible, or conditional risk of causing QT‐prolongation and/or TdP based on the AZCERT classification system [7]. The most prevalent individual and QTPMs combinations prescribed on discharge were identified, and subgroup analyses were performed to investigate differences in QTPMs and QTPM combinations between patients admitted to acute mental health units (AMHU) and other acute medical and geriatric units. Change in QTPM prescriptions between admission and discharge was evaluated with paired t‐tests using SPSS version 28.0.1.1. (IBM Corp. Released 2021. IBM SPSS Statistics for Windows, Version 28.0. Armonk, NY: IBM Corp). Significance was established at *p* < 0.05.

QTPMs doses prescribed to patients were evaluated by calculating the ratio of the prescribed daily dose (PDD) to the defined daily dose (DDD). The PDD was calculated as the dose multiplied by number of times the medication was taken per day. In the case of depot medications, the dose was divided by the number of days between administration. The DDD is defined by WHO [10] as the most common total daily dosage for a medication's most common indication. The PDD/DDD ratio had been used previously as a pragmatic way to inform relative doses between different medications [8]. The cut‐off for a drug to be considered ‘high dose’ was a PDD/DDD ratio ≥ 3.0. PDD/DDD > 3.0 are almost unequivocally above recommended doses in Australian guidelines [11]; with the notable exception of furosemide which has a wide dosing range depending on response. Hence, PDD/DDD ≥ 10 was considered a high dose for furosemide. Further details in eMethod and eTables 1–3. This study was approved by the human research ethics committees at the Department for Health and Wellbeing (HREC/16/SAH/130), and the institution (204486).

## 3 Results

The study population of 711 patients (eFigure 1) had a mean age of 54.7 years (SD = 21.0), were 52.0% female, and 37.4% in young to middle adulthood (eTable 4). The average length of hospitalization was 16.9 days (SD = 25.90) with 40.1% hospitalized for less than 1 week; 57.2% of included patients were admitted to acute mental health units (AMHU). The mean number of regular medications was 5.86 (SD = 4.31), and 6.10 (SD = 4.15) at admission and on discharge, respectively.

Compared to patients admitted to acute medical and geriatric units, patients admitted to AMHU were significantly younger (*p* < 0.001) and had significantly longer stays in hospital (*p* < 0.001) (Table 1). The latter were also prescribed significantly fewer medications both on admission (4.2 vs. 8.1, *p* < 0.001) and at discharge (4.6 vs. 8.1, both *p* < 0.001). The list of known, possible, and conditional QTPMs prescribed in the total cohort and top 20 drug–drug combinations and associated risk of QT prolongation for the two subgroups are shown in eTables 5–7 respectively.

**TABLE 1:** Study cohort demographics and prevalence of QTPMs.

Variable	Entire cohort (*n*=711)	Acute mental health units (*n*=407)	Acute medical and geriatric units (*n*=304)	*p* ^a^
Gender (%)				
Female	370 (52.0)^b^	205 (50.5)^b^	165 (54.3)	0.318
Male	340 (48.0)^b^	201 (49.5)^b^	139 (45.7)	
Age				
Mean (SD)	54.7 (21.0)	45.46 (17.6)	66.96 (18.9)	
18‐44, *n* (%)	266 (37.4)	223 (54.8)	43 (13.9)	< 0.00001^c^
45‐64, *n* (%)	197 (27.7)	122 (30.0)	75 (24.8)	
65 and above, *n* (%)	248 (34.9)	62 (15.2)	186 (61.4)	
Length of Stay, mean (SD)	16.9 (25.9)	19.9 (22.1)	12.9 (29.9)	0.00035
Mean number of regular medications (SD)				
Admission	5.9 (4.3)	4.2 (3.4)	8.1 (4.4)	< 0.00001
Discharge	6.1 (4.2)	4.6 (3.4)	8.1 (4.3)	< 0.00001
Prevalence of top 5 QTPMs prescribed, *n* (%)^d^				
Pre‐admission				
1	Quetiapine, 240 (33.8)	Quetiapine, 132 (32.4)	Quetiapine, 108 (35.5)	
2	Olanzapine, 150 (21.1)	Olanzapine, 94 (23.1)	Risperidone, 81 (26.6)	
3	Risperidone, 113 (15.9)	Aripiprazole, 59 (14.5)	Pantoprazole, 69 (22.7)	
4	Pantoprazole, 109 (15.3)	Paliperidone, 49 (12.0)	Olanzapine, 56 (18.4)	
5	Aripiprazole, 82 (11.5)	Mirtazapine, 43 (10.6)	Furosemide, 48 (15.8)	
Discharge				
1	Quetiapine, 200 (28.1)	Olanzapine, 106 (26.0)	Quetiapine, 96 (31.7)	
2	Olanzapine, 155 (21.8)	Quetiapine, 104 (25.6)	Risperidone, 76 (23.1)	
3	Pantoprazole, 108 (15.2)	Aripiprazole, 61 (15.0)	Pantoprazole, 63 (20.8)	
4	Risperidone, 103 (14.5)	Paliperidone, 54 (13.3)	Olanzapine, 49 (16.2)	
5	Aripiprazole, 86 (12.1)	Mirtazapine, 49 (12.0)	Furosemide, 42 (13.9)	
QTPMs prescribed for each patient				
Mean at admission (SD)	2.1 (1.1)	1.8 (0.9)	2.5 (1.1)	
Mean at discharge (SD)	2.0 (1.1)	1.9 (1.0)	2.3 (1.2)	
Observations	711	407	304	
Paired sample t‐statistic	−1.98	1.21	‐4.00	
*p* value (two‐tail)	0.05	0.227	< 0.001	
QTPMs on discharge: at least one known risk (KNW) in combination with conditional risk (CND) QTPMs, *n* (%)^d^				
Admission				
KNW + ≥ 1 CND	110 (15.5)	33 (8.1)	77 (25.3)	
KNW + ≥ 2 CND	44 (6.2)	9 (2.2)	35 (11.5)	
Discharge				
KNW + ≥ 1 CND	94 (13.2)	23 (5.7)	71 (23.4)	
KNW + ≥ 2 CND	36 (5.1)	6 (1.5)	30 (9.9)	

^a^ *p* values for the comparison of acute mental health unit vs. acute medical and geriatric units.

^b^ 1 patient was excluded as gender was not recorded.

^c^ *p* value for the comparison of the three age groups.

^d^ % calculated as proportion of patients taking each medicine/combination.

**FIGURE 1:**
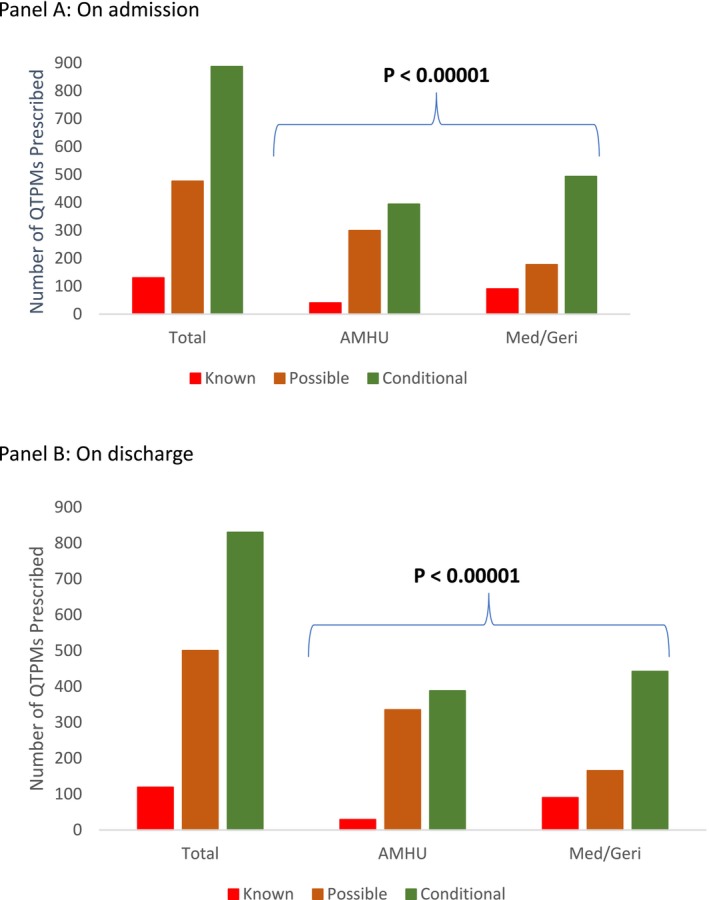
Distribution of QT‐prolonging medications among the study cohort.

The distribution of known, possible, and conditional QTPMs was significantly different between the two patient groups on admission and discharge (all *p* < 0.001) (Figure 1). Interestingly, patients admitted to AMHU were less likely to be prescribed medications with known QT prolonging risks [admission: 40 (5.5%) vs. 90 (11.8%); discharge: 29 (4.4%) vs. 90 (13.7%)] and more likely to be prescribed medications with possible risk of QT prolonging potential [admission: 299 (40.8%) vs. 177 (23.3%); discharge: 335 (42.2%) vs. 166 (22.8%)] when compared to those admitted to acute medical and geriatric units (both *p* < 0.001) (Figure 1). In the AMHU group, the five most frequently prescribed QTPMs were all antipsychotic medications, while at both time points, pantoprazole and furosemide were among the five most prescribed medications for the acute medical and geriatric unit group (Table 1).

The mean difference in the number of QTPMs decreased significantly between the two time points only in patients admitted to acute medical and geriatric units (2.5 to 2.3, *p* < 0.001) (Table 1). The mean cumulative PDD/DDD ratio for conditional risk QTPMs in the AMHU group was increased at discharge when compared to admission (mean at admission = 1.36 vs. at discharge = 1.56; *p* = 0.038) (eTable 8). Other dose comparisons between admission and discharge were not significant (eTable 8).

There were no significant differences between both groups when comparing the number of patients taking one QTPM with known risk in combination with one or more QTPMs with conditional risks at the two time points. However, when compared to patients admitted to AMHUs, patients admitted to acute medical and geriatric units were four and eight times more likely to be discharged with one QTPM with known risk in combination with more than one and two QTPMs with conditional risks, respectively (Table 1).

Even though high dose QTPMs were prescribed more often at discharge to patients admitted to AMHU, this difference was not significant (11.1% vs. 6.9%, *p* = 0.059). Logistic regression for patients prescribed high‐dose QTPMs at discharge showed a significant inverse relationship with age and a significant positive relationship with the total number of regular medicines at admission (eTable 9). This is indicative of older people being less likely to be prescribed high‐dose QTPMs and those who were admitted with more medications being more likely to be discharged with a high dose QTPM. However, the logistic regression model could only explain 8% of the variance (Nagelkerke *R* ^2^ = 0.078).

## 4 Discussion

This is the first Australian study to investigate the prescribing trends of and differences in QTPMs prescribing in patients admitted to AMHU and acute medical and geriatric units between admission and discharge. AMHU patients were more likely to be prescribed medications with possible QT‐prolonging potential, while patients admitted to acute medical and geriatric units were more likely to be discharged with combinations of medications with varying potential to cause QT prolongation.

It is possible that patients admitted to acute medical and geriatric units received a medication review during their hospital stay as they were significantly older and taking on average 8 regular medications [12, 13]. Even though ECG data was not available for analyses, it is possible that this group were more likely admitted for physical symptoms prompting the monitoring of ECGs, which would have resulted in monitoring of ECG and QTc. And the latter impacted the medication decision‐making process more so than in AMHU patients admitted with only psychological symptoms. However, the total number of regular medications in this group did not change significantly between admission and discharge despite the reduction in QTPMs (Table 1). Recent recognition of the prevalence of inappropriate antipsychotic and proton‐pump inhibitor (PPI‐ conditional QTPMs) use in the Australian elderly population has prompted changes to prescribing guidelines in this group. This may explain reduced prescribing of these medicines, especially in geriatric units [14, 15]. Despite this, 263 patients (64.6%) in AMHU and 229 patients (75.6%) admitted to acute medical and geriatric units were discharged with multiple QTPMs. Previous studies investigating polypharmacy in a geriatric psychiatric population and outpatients aged 65 years and older have reported similar results, with 71% and 59% of their populations respectively taking multiple QTPMs [16, 17].

Medications with conditional risk were prescribed more frequently than those with known or possible risk in both groups at both time points. While two‐drug combinations have been investigated, many patients were taking multiple drugs with some level of risk. For example, one AMHU patient was discharged with six QTPMs—three with possible and three with conditional risk as categorized by the AZCERT criteria [7]. As many of the drug–drug interactions identified have low levels of evidence to support the importance of these drug interactions (i.e., case report or theoretical) [18], future studies may identify patients on these combinations and monitor them closely to infer how the QT interval and risk of TdP change when complex medication regimens are used. Meid et al. [8] investigated associations between the QTc interval and combinations of drugs by their respective AZCERT category and reported additive QT prolongation with increasing numbers of known and conditional risk QTPMs but not for QTPMs with possible risk. This may signal that additional monitoring is warranted for the approximate 1 in 10 patients discharged from acute medical and geriatric units with a known risk QTPM co‐prescribed with two or more conditional risk QTPMs. Geriatric patients are also more likely to have other non‐medication related physical factors which may contribute to QT prolongation (e.g., electrolyte imbalances, cardiac ischemia) which may further increase their risk [19].

Exposure to higher doses of QTPMs has been observed to be correlated with QTc prolongation [8]. Approximately 9% of all patients in this study were discharged with QTPMs at higher than recommended doses. The increased doses of conditional risk QTPMs for AMHU patients at discharge were unsurprising given patients admitted to these units were, by definition, presenting with serious symptoms of mental illness. Management of these symptoms often requires higher doses of psychotropic medicines, and some of the most prescribed psychotropic medicines are conditional risk QTPMs (e.g., sertraline, olanzapine and quetiapine). The administration of high‐dose QTPMs should prompt close monitoring for QT prolongation while in hospital and post‐discharge. Post‐discharge follow‐up presents opportunities to reassess the need for high‐dose QTPMs in patients with stable mental illness. A multidisciplinary approach—through collaboration between psychiatrists, cardiologists, general practitioners, and pharmacists—to ECG monitoring and QTPM dose optimisation is recommended to manage these risks.

While QTPMs have been classified into three categories in this study, it should be noted that each medication would contribute varying degrees to the overall prolongation of the QT interval and relative risk of TdP. At therapeutic doses, QTc prolongation can range from 4.7 ms with haloperidol (known risk) and 6.4 ms with olanzapine (conditional risk) to 20.6 ms with ziprasidone (conditional risk) [20]. Roden [21] suggested the relationship between the extent of QT‐prolongation and the risk of developing TdP is non‐linear, even though this relationship appeared to be positively correlated. It is an imperfect surrogate marker; however, prescribers should be aware that patients prescribed any drug that prolongs the QT interval are at a greater risk of TdP and sudden cardiac death ([22, 23]).

Clinical rationale for drug choices was not available as individual patient case notes were not available for review. The possibility of pre‐existing QT abnormalities on medication choices could not be inferred as ECGs were not routinely performed on admission and following QTPM initiation. Further, short‐term use of QTPM during hospitalization only, which may increase TdP risk, was not assessed in this study. Current research suggests the co‐prescribing of drugs with a risk of QT prolongation and TdP is one of many factors that impact the risk of developing these conditions [24, 25]. As ECGs were not routinely carried out at admission or after initiation of a drug, we were unable to infer whether prescribers were taking the overall risk of developing QT prolongation into account when prescribing new medicines.

The prescribing trends identified in this study should prompt further research into evaluating the risk of developing QT prolongation and TdP in different patient groups. Access to ECG data, electrolyte levels, and comorbidities could assist in characterizing how individual medicines contribute to the overall risk of QT prolongation. Pharmacokinetic drug interactions should also be included in future research to identify other high‐risk medications. Validation of studies such as Meid et al. [8] will provide a better understanding of how drug combinations affect the QT interval and the risk of TdP. The difference in QT prolonging potential and risk of TdP between medications in the same AZCERT risk category warrants more specific quantification of risk potential to be developed. Investigating the incidence of patients discharged on multiple QT‐prolonging medications who are re‐hospitalized with heart conditions or experience sudden cardiac death could provide guidance as to whether this risk is being identified in current practice or if it requires further attention.

## Author Contributions

Study concept and design: Elizabeth Hotham, Sepehr Shakib, Vijayaprakash Suppiah. Acquisition of data: Sepehr Shakib. Analysis and interpretation of data: Joel Moore, Isabella Singh, Ruby Tszwai Au, Vijayaprakash Suppiah. Drafting of the manuscript: Joel Moore, Isabella Singh, Genevieve Gabb, Joanne Eng‐Frost, Sepehr Shakib, Vijayaprakash Suppiah. Critical revision of the manuscript for important intellectual content: Joel Moore, Isabella Singh, Ruby Tszwai Au, Genevieve Gabb, Joanne Eng‐Frost, Elizabeth Hotham, Sepehr Shakib, Vijayaprakash Suppiah.

## Ethics Statement

This study was approved by the human research ethics committees at the Department for Health and Wellbeing (HREC study number: HREC/16/SAH/130) and the University of South Australia (Protocol no: 204486).

## Conflicts of Interest

The authors declare no conflicts of interest.

## Supporting information


Data S1.

## Data Availability

The datasets analyzed during the current study are available from the corresponding author on reasonable request.
