# Supplementary material for: Psychotropic Polypharmacy and QT Prolonging Medications in Hospitalized Patients
Source: Pharmacol Res Perspect. 2025 Apr 29;13(3):e70107. doi: 10.1002/prp2.70107 (PMC12041124; doi:10.1002/prp2.70107)
Supplement: Supplementary file 1 — Data S1. [file PRP2-13-e70107-s001.docx]

**Supplemental online content**

eMethods. Methodology

eTable 1. Inclusion and exclusion criteria

eTable 2. Variables derived from patients’ parameters for descriptive and exploratory analyses

eTable 3. Summary of assumptions made for discharge medications

eFigure 1. The process of patient selection and exclusion

eTable 4. Demographics of the study population

eTable 5. List of all QTPMs prescribed in the total cohort according to their AZCERT risk categories

eTable 6. Top 20 QT-prolonging discharge drug combinations for patients admitted to acute mental health units

eTable 7. Top 20 QT-prolonging discharge drug combinations for patients admitted to acute medical and geriatric units

eTable 8. Count and mean cumulative PDD/DDD ratio by AZCERT risk classification

eTable 9. Logistic regression for high dose QTPMs coefficient significance

**eMethods. Methodology**

**Study population**

The present study included patients admitted to a metropolitan acute care teaching hospital, a metropolitan hospital with a range of services including 24/7 community emergency department (ED) and inpatient mental health facilities, and a regional acute care hospital with 70 beds. The de-identified data included medication information such as medication name, dosage, route of administration, prescription status (pre-admission, during hospital stay, or on discharge), frequency and the type of medication (regular or pro re nata (PRN)), and patient data such as patients’ visit type, admission and discharge dates and times, admission unit, discharge status, age and gender.

The inclusion criteria for this study were patients aged 18 and above, admitted to an inpatient unit or emergency department and whose medication history had been documented or verified by a hospital pharmacist. Patients who were admitted for less than 48 hours were excluded, as a 48 hours period was considered to be insufficient for prescribers to make changes to the patients’ medications. Patients were only included if they were taking regular antipsychotic/s prior to admission with documentation of the dosage. Therefore, patients were excluded if they were only taking PRN antipsychotic medications prior to admission, or only taking antipsychotics during their hospital stay or on discharge. Additionally, patients admitted to palliative care, surgical, or oncology units were excluded. Patients not discharged from the hospitals were also excluded, as outlined in the inclusion and exclusion criteria below (eTable 1).

**eTable 1. Inclusion and exclusion criteria**

| Inclusion criteria | Exclusion criteria |
| --- | --- |
| - Aged 18 years and above - Patients admitted to the inpatient unit or emergency department - Use of at least one regular antipsychotic medication on admission with dosage documented - Length of hospitalisation > 48 hours - Patients whose medication history has been documented or verified by a hospital pharmacist | - Patients admitted to   - Palliative care units   - Surgical units   - Oncology units - Patients transferred to another hospital or who died during their hospital stay - Patients that became maintenance patients |

Additional patient variables were available in the dataset for descriptive and exploratory analyses (eTable 2). For example, there are three categories for “visit type”, inpatient and emergency department were documented in the dataset, while the third group recorded as “transferred from the emergency department to inpatient units” was derived from the patients’ information. This group of patients was identified by the overlapped emergency department discharge date and time with the inpatient admission date and time. Another example is the length of stay. The length of hospitalisation was defined by the date and time between admission and discharge for each patient. Where the difference between the two-time points was not a full day, the hours and minutes were converted to days. For example, 18 days, 23 hours, 15 minutes = 18.97 days. For descriptive and stratified analyses, the continuous variable was converted to ordinal dummy-coded variables for a better explanation of the findings. It was categorised into three groups: patients hospitalised for one week or less, hospitalised for more than a week to 15 days, and more than 15 days. Further, continuous variables such as age and the mean number of medications were converted into ordinal variables for stratified analyses. According to the Australian Bureau of Statistics (ABS), there are three categories of the international age classifications, with the lowest level consisting of six groups [1]. In accordance with the ABS, patients in the present study were divided into three categories: young and middle adulthood (18-44), older adulthood (45-64), and elderly (65 and over).

**eTable 2. Variables derived from patients’ parameters for descriptive and exploratory analyses**

| Variables | Descriptive analysis | Exploratory analysis |
| --- | --- | --- |
| Visit type | Nominal: Additional group was identified by the overlapped emergency department discharge date and time with the inpatient admission date and time, and emergency departments (n=5) was analysed as part of the “transferred from the emergency department to inpatient units” (n=129) due to the considerably small sample size | |
| Length of stay (LOS) | Ordinal:  2<LOS<7, 7≤LOS<15, LOS≥15 | Continuous: Calculated from the differences between admission and discharge dates and times |
| Units | Nominal: Emergency departments (n=5) were analysed as part of the acute medical units (n=61) due to the considerably small sample size | |
| Age | Ordinal:  18-44, 45-64, 65 and above | Continuous |
| Mean number of medications | Ordinal:  Less than five regular medications, and five or more regular medications | Continuous: Average of the regular medications taken on admission and discharge |

**Assumptions for discharge medications**

Assumptions were made according to the medications codes to determine the discharge medications. All medications in the dataset were coded against one of three classifications: HISA, DISCD and AUA1. These correspond to medications the patients were taking prior to admission (HISA), medications that were prescribed during hospitalisation and were discontinued automatically upon discharge (DISCD), and medications that were prescribed on discharge (AUA1).

To ensure consistency in the interpretation of the data, the following assumptions were made in determining whether the medications were given upon discharge, and their dose and frequency (eTable 3):

1. For medications with a status of AUA1 (prescribed and supplied on discharge medications), the documented dose and frequency was the AUA1 dose and frequency
2. For medications with both statuses DISCD (discontinued on discharge medications) and AUA1 (prescribed and supplied on discharge medications), their documented doses and frequencies were the AUA1 doses and frequencies
3. For medications with both statuses HISA (pre-admission medications) and DISCD (discontinued on discharge medications), it was assumed that these medications were continued upon discharge and their documented doses and frequencies would be the same as the DISCD doses and frequencies (that is, what the patients were taking during their hospital stay)
4. For medications with a status of HISA (pre-admission medications) only, it is assumed that these medications were discontinued
   1. Exception: the medication is a depot medication with no equivalent prescribed as DISCD or AUA1
5. For medications with a status of DISCD (discontinued on discharge medications) only, it is assumed that these medications were discontinued
   1. Exception: the medication is a depot medication with no equivalent prescribed as AUA1

**eTable 3.** **Summary of assumptions made for discharge medications**

|  | HISA | DISCD | AUA1 | Continued on discharge | Dose and frequency |
| --- | --- | --- | --- | --- | --- |
| Assumption 1 |  |  | ✓ | YES | AUA1 |
| Assumption 2 |  | ✓ | ✓ | YES | AUA1 |
| Assumption 3 | ✓ | ✓ | ☓ | YES | DISCD |
| Assumption 4 | ✓ | ☓ | ☓ | NO | N/A |
| Assumption 5 | ☓ | ✓ | ☓ | NO | N/A |

*Note*. Medications with the specific order status (HISA/DISCD/AUA1) are indicated by “✓”, medications without the specific order status are indicated by “☓”, boxes coloured in grey indicate that the status of the medication will not impact the outcome of the assumptions

These algorithms were developed based on the following assumptions. If a patient was given medication with a code of DISCD, it was assumed that the medication was initiated during the patient's hospital stay and discontinued upon discharge. However, if the medication had both HISA and DISCD codes, it was assumed that the patient was taking this medication prior to admission and was also given this medication during the patient's hospital stay. Hence, it was unlikely that the medication was stopped abruptly on discharge. The reasons for the missing AUA1 code could be that the patient was taking the medication regularly and did not require an additional Pharmaceutical Benefits Scheme (PBS) script for additional units. However, it is also possible that this medication could have been a long-acting medication, such as a depot injection.

**eFigure 1. The process of patient selection and exclusion**

Patients included after preliminary screening

(n=2,715)

Patients taking antipsychotics prior to admission (n=1,038)

Patients not taking antipsychotics prior to admission (n=1,677)

Patients included

(n=711)

Excluded^a^:

- Length of stay less than 48 hours (n=156)
- Patients taking PRN antipsychotics (n=41)
- Patients taking pre-admission sedating medications with missing dosage information (n=144)
- Patients admitted to oncology units (n=5)
- Patients that became maintenance patients (n=3)

^a^For patients with multiple admissions, only the first admission that satisfied the inclusion criteria was included. Hence, the number of patients excluded with reasons does not add up to the total number of patients excluded.

**eTable 4. Demographics of the study population**

| Characteristics | Total (n=711) |
| --- | --- |
| Female, n (%) ^a^ | 370 (52.0) |
| Age |  |
| Mean (SD) | 54.65 (21.04) |
| 18-44, n (%) | 266 (37.4) |
| 45-64, n (%) | 197 (27.7) |
| 65 and above, n (%) | 248 (34.9) |
| Number of regular medications, mean (SD) |  |
| Total | 5.99 (4.04) |
| Pre-admission | 5.86 (4.31) |
| Discharge | 6.10 (4.15) |
| Length of stay |  |
| Mean (SD) | 16.87 (25.90) |
| 2≤LOS<7, n (%) | 285 (40.1) |
| 7≤LOS<15, n (%) | 207 (29.1) |
| LOS≥15, n (%) | 219 (30.8) |
| Facility |  |
| Facility A, n (%) | 520 (73.1) |
| Facility B, n (%) | 147 (20.7) |
| Facility C, n (%) | 44 (6.2) |
| Unit ^a^ |  |
| Acute Mental Health, n (%) | 407 (57.2) |
| General Medical, n (%) | 105 (14.8) |
| Medical Subspecialties, n (%) | 67 (9.4) |
| Acute medical, n (%) | 66 (9.3) |
| Geriatric, n (%) | 65 (9.1) |
| Visit type |  |
| Inpatient, n (%) | 577 (81.2) |
| ED to inpatient, n (%) | 134 (18.8) |
| Discharge Location ^a^ |  |
| Home, n (%) | 579 (81.4) |
| Residential Care Facility, n (%) | 131 (18.4) |

^a^ Missing data n=1

**eTable 5.** **List of all QTPMs prescribed in the total cohort according to their AZCERT risk categories**

| **QTPMs prescribed within the cohort by AZCERT risk category** | | |
| --- | --- | --- |
| ***Known risk*** | ***Possible risk*** | ***Conditional risk*** |
| amiodarone | aripiprazole | amisulpride |
| azithromycin | asenapine | amitriptyline |
| chlorpromazine | atomoxetine | amphotericin B |
| ciprofloxacin | bicalutamide | clomipramine |
| citalopram | buprenorphine | diltiazem |
| clarithromycin | clozapine | doxepin |
| domperidone | flupentixol | esomeprazole |
| donepezil | levetiracetam | famotidine |
| droperidol | lithium carbonate | fluoxetine |
| escitalopram | lurasidone | fluvoxamine |
| flecainide | mirabegron | furosemide (frusemide) |
| haloperidol | mirtazapine | galantamine |
| methadone | nortriptyline | hydrochlorothiazide |
| moxifloxacin | paliperidone | indapamide |
| ondansetron | promethazine | ivabradine |
| roxithromycin | tamoxifen | lansoprazole |
| sotalol | tramadol | loperamide |
|  | venlafaxine | metoclopramide |
|  | zuclopenthixol | metronidazole |
|  |  | olanzapine |
|  |  | omeprazole |
|  |  | pantoprazole |
|  |  | paroxetine |
|  |  | quetiapine |
|  |  | risperidone |
|  |  | sertraline |
|  |  | solifenacin |
|  |  | ziprasidone |

**eTable 6.** **Top 20 QT-prolonging discharge drug combinations for patients admitted to acute mental health units^a^**

|  | **Name** | **Risk of causing QT-prolongation and TdP** | |  |
| --- | --- | --- | --- | --- |
| **Rank** | **Medication 1-Medication 2** | **Medication 1** | **Medication 2** | **n^b^** |
| 1 | Mirtazapine-olanzapine | Possible | Conditional | 20 |
| 2 | Lithium-quetiapine | Possible | Conditional | 19 |
| 3 | Lithium-olanzapine | Possible | Conditional | 17 |
| 4 | Pantoprazole-quetiapine | Conditional | Conditional | 17 |
| 5 | Mirtazapine-quetiapine | Possible | Conditional | 16 |
| 6 | Olanzapine-pantoprazole | Conditional | Conditional | 13 |
| 7 | Olanzapine-paliperidone | Conditional | Possible | 12 |
| 8 | Aripiprazole-quetiapine | Conditional | Conditional | 12 |
| 9 | Quetiapine-venlafaxine | Conditional | Possible | 11 |
| 10 | Aripiprazole-olanzapine | Conditional | Conditional | 10 |
| 11 | Aripiprazole-mirtazapine | Conditional | Possible | 10 |
| 12 | Lithium-mirtazapine | Possible | Possible | 10 |
| 13 | Olanzapine-zuclopenthixol | Conditional | Possible | 8 |
| 14 | Paliperidone-quetiapine | Possible | Conditional | 8 |
| 15 | Escitalopram-quetiapine | Known | Conditional | 8 |
| 16 | Mirtazapine-pantoprazole | Possible | Conditional | 8 |
| 17 | Olanzapine-quetiapine | Conditional | Conditional | 7 |
| 18 | Quetiapine-sertraline | Conditional | Conditional | 7 |
| 19 | Lithium-paliperidone | Possible | Possible | 7 |
| 20 | Mirtazapine-venlafaxine | Possible | Possible | 7 |

^a^ Table shows only 2-drug combinations, if a patient is taking >2, for example mirtazapine-olanzapine-lithium, this is counted as three separate combinations (mirtazapine-olanzapine, olanzapine-lithium and lithium-mirtazapine).

^b^ Out of 483 total two-drug combinations

**eTable 7. Top 20 QT-prolonging discharge drug combinations for patients admitted to acute medical and geriatric units ^a^**

|  | **Name** | **Risk of causing QT-prolongation and TdP** | |  |
| --- | --- | --- | --- | --- |
| **Rank** | **Medication 1–Medication 2** | **Medication 1** | **Medication 2** | **n**^b^ |
| 1 | Pantoprazole-risperidone | Conditional | Conditional | 19 |
| 2 | Escitalopram-risperidone | Known | Conditional | 18 |
| 3 | Furosemide-risperidone | Conditional | Conditional | 17 |
| 4 | Pantoprazole-quetiapine | Conditional | Conditional | 15 |
| 5 | Buprenorphine-risperidone | Possible | Conditional | 14 |
| 6 | Furosemide-pantoprazole | Conditional | Conditional | 14 |
| 7 | Mirtazapine-quetiapine | Possible | Conditional | 12 |
| 8 | Olanzapine-pantoprazole | Conditional | Conditional | 12 |
| 9 | Esomeprazole-quetiapine | Conditional | Conditional | 11 |
| 10 | Aripiprazole-pantoprazole | Conditional | Conditional | 10 |
| 11 | Escitalopram-quetiapine | Known | Conditional | 8 |
| 12 | Furosemide-quetiapine | Conditional | Conditional | 8 |
| 13 | Citalopram-quetiapine | Known | Conditional | 8 |
| 14 | Mirtazapine-risperidone | Possible | Conditional | 8 |
| 15 | Escitalopram-pantoprazole | Known | Conditional | 8 |
| 16 | Omeprazole-quetiapine | Conditional | Conditional | 7 |
| 17 | Omeprazole-risperidone | Conditional | Conditional | 7 |
| 18 | Mirtazapine-pantoprazole | Possible | Conditional | 7 |
| 19 | Buprenorphine-pantoprazole | Possible | Conditional | 7 |
| 20 | Aripiprazole-furosemide | Conditional | Conditional | 7 |

^a^ Table shows only 2-drug combinations, if a patient is taking >2, for example mirtazapine-olanzapine-lithium, this is counted as three separate combinations (mirtazapine-olanzapine, olanzapine-lithium and lithium-mirtazapine).

^b^ Out of 612 total two-drug combinations

**eTable 8. Mean cumulative PDD/DDD ratio by AZCERT risk classification within group comparison at admission vs. discharge**

Mean cumulative PDD/DDD ratio was calculated as follows:

$$Mean cumulative\frac{PDD}{DDD}ratio = \left( \frac{{PDD}_{1}}{{DDD}_{1}} \right)+ \left( \frac{{PDD}_{2}}{{DDD}_{2}} \right)+ \left( \frac{{PDD}_{3}}{{DDD}_{3}} \right)\ldots$$

|  | AZCERT risk category | Admission cumulative PDD/DDD ratio, mean (SD) | Discharge cumulative PDD/DDD ratio, mean (SD) | p-value for independent t-tests |
| --- | --- | --- | --- | --- |
| Entire cohort ^a^ | Known | N = 120  1.39 (0.99) | N = 112  1.34 (0.90) | 0.702 |
|  | Possible | N = 390  1.44 (1.00) | N = 392  1.53 (1.06) | 0.224 |
|  | Conditional | N = 569  1.49 (1.33) | N = 545  1.60 (1.39) | 0.173 |
| Acute mental health units ^a^ | Known | N = 39  1.48 (0.99) | N = 29  1.45 (0.84) | 0.875 |
|  | Possible | N = 244  1.63 (1.02) | N = 254  1.78 (1.10) | 0.138 |
|  | Conditional | N = 294  1.36 (1.17) | N = 288  1.56 (1.17) | **0.038** |
| Acute medical and geriatric units^a^ | Known | N = 81  1.34 (0.99) | N = 83  1.31 (0.92) | 0.769 |
|  | Possible | N = 146  1.12 (0.87) | N = 138  1.08 (0.84) | 0.714 |
|  | Conditional | N = 275  1.63 (1.47) | N = 257  1.64 (1.59) | 0.906 |

^a^ includes only individuals who are prescribed at least one medication in the respective AZCERT class

**eTable 9. Logistic regression for high dose QTPMs coefficient significance**

| **Categorical Variables Codings** | | | |
| --- | --- | --- | --- |
|  | | Frequency | Parameter coding |
|  |  |  |  |
| Gender | Male | 340 | 0 |
|  | Female | 370 | 1 |
| VisitType | Inpatient | 577 | 0 |
|  | Emergency | 133 | 1 |
| Admitting unit | AMHU | 407 | 0 |
|  | AMGU | 304 | 1 |

| **Variables in the Equation** | | | | | | | |
| --- | --- | --- | --- | --- | --- | --- | --- |
|  | | B | S.E. | Wald | df | Sig. | Exp(B) |
|  | Number of regular medications at admission | .143 | .032 | 20.165 | 1 | **<.001** | 1.154 |
|  | Age | -.025 | .009 | 8.315 | 1 | **.004** | .975 |
|  | VisitType (1) | .327 | .326 | 1.009 | 1 | .315 | 1.387 |
|  | Gender (1) | -.206 | .269 | .587 | 1 | .443 | .814 |
|  | Length of stay (number of days) | .003 | .006 | .191 | 1 | .662 | 1.003 |
|  | Admitting unit (1) | -.545 | .349 | 2.442 | 1 | .118 | .580 |
|  | Constant | -1.691 | .404 | 17.566 | 1 | <.001 | .184 |
|  | | | | | | | |

References:

1. Australian Bureau of Statistics 2014, *Age Standard*, viewed 6 July 2021, <<https://www.abs.gov.au/statistics/standards/age-standard/latest-release>>.

2. World Health Organization 2019, *Medication Safety in Polypharmacy*, World Health Organization, Switzerland. <<https://www.who.int/publications/i/item/medication-safety-in-polypharmacy-technical-report>>.

3. Hilmer, S.N., et al. 2007, 'A drug burden index to define the functional burden of medications in older people', *Archives of Internal Medicine,* vol*.* 167, no. 8, pp. 781-787.

4. Therapeutic Goods Administration 2021, *Product and Consumer Medicine Information*, Australian Government Department of Health, viewed 17 August 2021, <<https://www.ebs.tga.gov.au/>>.

5. Australian Medicines Handbook (online) 2021, Australian Medicines Handbook Pty Ltd, Adelaide, viewed 20 August 2021, <<https://amhonline.amh.net.au/>>.

6. O’Connell, J. 2020, 'Addressing the Complexity of Medication Use in Older People with Intellectual Disability', Doctor of Philosophy thesis, School of Pharmacy and Pharmaceutical Sciences, University of Dublin, Trinity College. <<http://www.tara.tcd.ie/bitstream/handle/2262/90948/PhD%20Thesis%20Minor%20Corrections%20Plain%20Text%20Version%202020.pdf?sequence=3>>.

7. Bostock, C.V., R.L. Soiza, and A.A. Mangoni 2013, 'Associations between different measures of anticholinergic drug exposure and Barthel Index in older hospitalized patients', *Therapeutic Advances in Drug Safety,* vol*.* 4, no. 6, pp. 235-245.

8. Gnjidic, D., et al. 2012, 'Drug Burden Index associated with function in community-dwelling older people in Finland: a cross-sectional study', *Annals of Medicine,* vol*.* 44, no. 5, pp. 458-467.

9. Jamieson, H.A., et al. 2019, 'Drug burden index and its association with hip fracture among older adults: a national population-based study', *The Journals of Gerontology: Series A,* vol*.* 74, no. 7, pp. 1127-1133.

10. Hilmer, S.N. 2018, 'Calculating and using the drug burden index score in research and practice', *Expert Review of Clinical Pharmacology,* vol*.* 11, no. 11, pp. 1053-1055.
